# 3-(2-Chloro-3-hy­droxy-4-meth­oxy­phen­yl)-1-(4,5-dimeth­oxy-2-methyl­phen­yl)prop-2-en-1-one

**DOI:** 10.1107/S1600536812038275

**Published:** 2012-09-12

**Authors:** U. H. Patel, S. A. Gandhi, V. M. Barot, M. C. Patel

**Affiliations:** a Department of Physics, Sardar Patel University, Vallabh Vidya Nagar, Gujarat 388 120, India; b P. G. Center in Chemistry, Smt. S. M. Panchal Science College, Talod, Gujarat 383 215, India

## Abstract

The title compound, C_19_H_19_ClO_5_, is a chloro derivative of a biologically significant chalcone family. The mean plane of the two substituted benzene rings are twisted by 55.33 (8)° with respect to each other. An intra­molecular C—H⋯Cl hydrogen bond generates an *S*(5) graph-set motif. In the crystal, a bifurcated O—H⋯(O,O) hydrogen bond leads to an *R*_1_^2^(5) graph-set motif and to the formation of zigzag chains propagating along the *c*-axis direction. A weak π–π inter­action involving the methyl­phenyl rings [centroid–centroid distance = 3.8185 (10) Å] and C—H⋯π inter­actions also occur.

## Related literature

For the biological activity of chalcones, see: Awasthi *et al.* (2009 ▶); Cheng *et al.* (2000 ▶); Echeverria *et al.* (2009 ▶); Szliszka *et al.* (2010 ▶); Yadav *et al.* (2010 ▶); Bhatia *et al.* (2009 ▶); Lahtchev *et al.* (2008 ▶); Yayli *et al.* (2006 ▶); Sivakumar *et al.* (2010 ▶). For our studies on the synthesis and crystal structures of chalcones, see: Patel *et al.* (2007*a* ▶,*b* ▶). For C—H⋯π inter­actions, see: Malone *et al.* (1997 ▶); For standard bond lengths, see: Allen *et al.* (1987 ▶). For hydrogen-bond motifs, see: Bernstein *et al.* (1995 ▶).
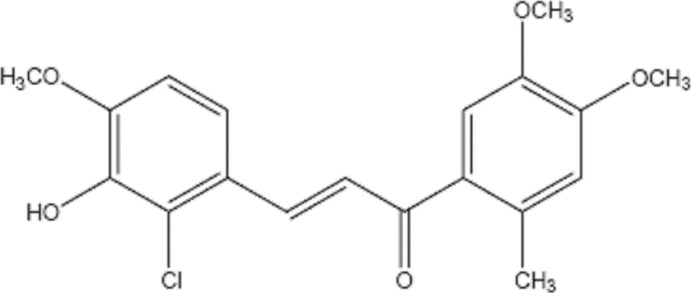


## Experimental

### 

#### Crystal data

C_19_H_19_ClO_5_
*M* *_r_* = 362.79
Orthorhombic, 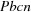

*a* = 14.2134 (4) Å
*b* = 10.2802 (2) Å
*c* = 24.2786 (7) Å
*V* = 3547.51 (16) Å^3^
*Z* = 8
Mo *K*α radiation
μ = 0.24 mm^−1^
*T* = 293 K
0.32 × 0.21 × 0.07 mm

#### Data collection

Bruker Kappa APEXII CCD diffractometer
34815 measured reflections
3135 independent reflections
2399 reflections with *I* > 2σ(*I*)
*R*_int_ = 0.040

#### Refinement

*R*[*F* ^2^ > 2σ(*F* ^2^)] = 0.034
*wR*(*F* ^2^) = 0.098
*S* = 1.05
3135 reflections
266 parameters
H atoms treated by a mixture of independent and constrained refinement
Δρ_max_ = 0.16 e Å^−3^
Δρ_min_ = −0.21 e Å^−3^

### 

Data collection: *APEX2* (Bruker, 2008 ▶); cell refinement: *SAINT* (Bruker, 2008 ▶); data reduction: *SAINT*; program(s) used to solve structure: *SHELXS97* (Sheldrick, 2008 ▶); program(s) used to refine structure: *SHELXL97* (Sheldrick, 2008 ▶); molecular graphics: *PLATON* (Spek, 2009 ▶); software used to prepare material for publication: *publCIF* (Westrip, 2010 ▶).

## Supplementary Material

Crystal structure: contains datablock(s) I, global. DOI: 10.1107/S1600536812038275/zj2087sup1.cif

Structure factors: contains datablock(s) I. DOI: 10.1107/S1600536812038275/zj2087Isup2.hkl

Supplementary material file. DOI: 10.1107/S1600536812038275/zj2087Isup3.cml

Additional supplementary materials:  crystallographic information; 3D view; checkCIF report

## Figures and Tables

**Table 1:** Hydrogen-bond geometry (Å, °) *Cg*1 and *Cg*2 are the centroids of the C1–C6 and C11–C16 rings, respectively.

*D*—H⋯*A*	*D*—H	H⋯*A*	*D*⋯*A*	*D*—H⋯*A*
O1—H11⋯O4^i^	0.84 (3)	2.31 (3)	3.007 (2)	140 (3)
O1—H11⋯O5^i^	0.84 (3)	2.37 (3)	3.042 (2)	138 (2)
C8—H8⋯Cl1	0.95 (2)	2.62 (2)	3.044 (2)	107.9 (15)
C7—H72⋯*Cg*2^i^	0.96	2.92	3.720 (3)	142
C18—H181⋯*Cg*1^ii^	0.96	3.00	3.512 (2)	115

Symmetry codes: (i) 
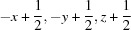
; (ii) 
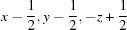
.

**Fig. 1.**
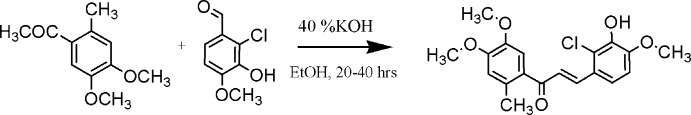
Reaction Scheme for C19 H19 Cl O5.

**Fig. 2.**
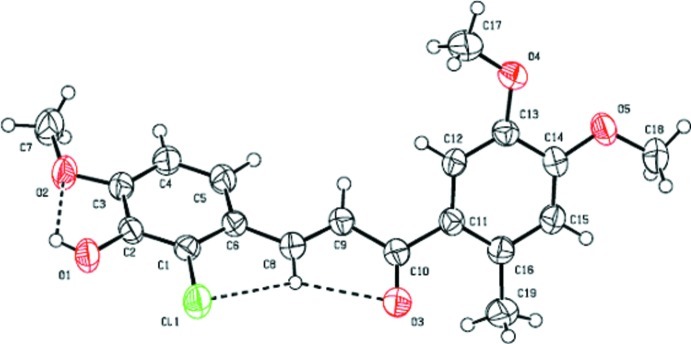
Molecular structure of the title compound,showing the atom labeling scheme with 50% probability displacement ellipsoids.

**Fig. 3.**
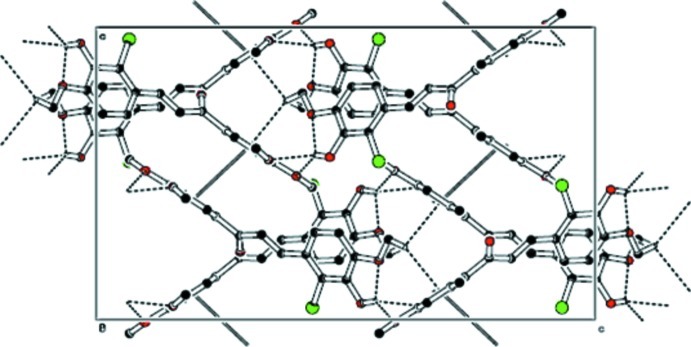
Packing diagram of the title molecule, showing trifurcated O—H···O interactions, C—H···π and π-π interactions on ac plane.

## supplementary crystallographic information

### Comment

Chalcones, belonging to flavonoid family, synthesized or the natural one, displayed many interesting properties including antimalarial (Awasthi *et al.*, 2009; Cheng *et al.*, 2000), anticancer (Echeverria *et al.*, 2009 Szliszka *et al.*, 2010), anti-inflammatory (Yadav *et al.*, 2010), antibacterial (Bhatia *et al.*,2009), antifungal (Lahtchev *et al.*,2008), antimicrobial (Yayli *et al.*,2006) and antioxidant (Sivakumar *et al.*, 2010) activities. In the chemical structure, three carbon α – β unsaturated carbonyl system, the back bone of the open chain flavonoids, joins two aromatic rings. In view of the pharmacological importance of chalcones and in continuation of our interest on the synthesis and crystal structure determination of interesting class of heterocyclic compounds (Patel *et al.*, 2007*a*,*b*), we report here the synthesis and crystal structure of newly synthesized methoxy – chloro substituted C_19_H_19_ClO_5_ chalcone derivative.

In the title compound, C_19_H_19_ClO_5_ (I), two methoxy and one methyl groups present in one of the phenyl rings which is joined by a prop-2-en-1-one group to chloro – hydroxyl- methoxy substituted phenyl ring. Bond distances are at normal range (Allen *et al.*, 1987). The dihedral angle between the mean plane of the two benzene rings is 55.33 (8)° and the angle between the mean plane of prop-2-en 1-one group (C8\C9\O3\C10) and the mean plane of the Chloro phenyl ring (C1\C2\C3\C4\C5\C6) and methyl phenyl ring (C11\C12\C13\C14\C15\C16) are 21.85 (12)° and 34.09 (12)° respectively.

In terms of graph set analysis (Bernstein *et al.*, 1995), intra molecular interactions O1—H11···O2, C8—H8···O3 and C8—H8···Cl1 generate three pseudo rings of S(5) graph set motifs (Fig. 2). The O1—H11···O4 (-*x* + 1/2,-*y* + 1/2,*z* + 1/2) and O1—H11···O5 (-*x* + 1/2,-*y* + 1/2,*z* + 1/2) interactions form a pair of bifurcated donor bonds involving methoxy oxygen atoms O2 and O4 of the symmetry related molecule at -*x* + 1/2,-*y* + 1/2,*z* + 1/2 generating a ring of graph set motif *R*_1_^2^ (5) which form a zig - zag molecular chain running parallal to the [0 0 1] direction. This molecular arrangement facilitates in formation of a C—H···π interaction of type - III (Malone *et al.* (1997)) involving the centroids of the methyl phenyl ring to methoxy carbon C7 *via* H72. Another C—H···π interaction of type - III involves methoxy carbon C18 *via* H18 to the centroid of chloro phenyl ring. The weak π–π stacked interaction involving the centroids of the methyl phenyl ring with Cg–Cg separation distance of 3.8185 (10) Å further contributes to the molecular packing (Fig. 3). Superimposed symmetry related molecules connected by trifurcated O—H···O hydrogen bonds, C—H···Cl, C—H···O and π–π interactions lined up parallel to [0 0 1] direction.

### Experimental

Preparation of 1-(4, 5-dimethoxy- 2- methyl-phenyl) ethanone (0.01 mole) and 2-chloro-3-hydroxy-4-methoxy benzaldehyde (0.01 mole) were dissolved in ethanol (40 ml) and a solution of potassium hydroxide (40%, 40 ml) was added in it (Fig. 1). The reaction mixture was stirred at room temperature for 24 h. After completion of the reaction as indicated by TLC, contents were poured in to crushed ice and acidified with diluted HCl. The solid separated was washed with water, filtered and dried. Yield: 89%, Elemental Analysis: C – 63.40%, H – 6.12%, Cl-9.30% IR(cm-1): 2956(C—H str. (*asym*)alkyl), 2893 (C—H str. (*sym*) alkyl), 1486 (C-Hdef. (*asym*) alkyl), 1377 (C—H def. (*sym*) alkyl), 3048 (C—H str.arom.), 1588, 1528.(C=C str. arom.), 1118 (C—H i.p.def arom.), 819 (C—H o.o.p.def.arom.), 3373(OH, phenol), 1278 (C—O—C (*sym*)ether), 1081(C—O—C (*asym*) ether), 985 (CH=CH def.chalcone), 3042(CH=CH str. chalcone), 1629 (C=C str. chalcone), 506 (C—Cl str.). 1H NMR (CDCl_3_) p.p.m.: 2.44(s, 3*H*, CH_3_), 3.89(s, 3*H*, OCH_3_), 3.93(s, 3*H*, OCH_3_), 3.94(s, 3*H*, OCH_3_), 6.74(s, 1*H*), 6.94(s, 1*H*), 7.06(d, 2*H*, J = 7.2 Hz), 7.45(d,1*H*,J=15.4 Hz,chalcone), 7.61(d, 1*H*, J = 15.6 Hz, chalcone), 13.46(s, OH).MS: m/*z* 365(*M*+2). ^13^C NMR (CDCl_3_) p.p.m.: 15.38(CH_3_), 55.79(OCH_3_), 56.01(OCH_3_), 56.04(OCH_3_), 122.75(CH=CH, chalcone), 126.31(CH=CH,chalcone), 146.25(C—Cl), 155.27(C—OH), 194.56(C=O).

### Refinement

The H atoms positions are geometrically fixed. These H atoms are constrained to ride on their parent atoms with *U*_iso_(H) = 1.2*U*_eq_(C) for the phenyl H atoms and *U*_iso_(H) = 1.5*U*_eq_(C) for the methyl H atoms. Data collection: *APEX2* (Bruker, 2008) Cell refinement: *SAINT* (Bruker, 2008) Data reduction: *SAINT* Program(S) used to solve structure: *SHELXS97* (Sheldrick, 2008) Program(S) used to refine structure: *SHELXL97*(Sheldrick, 2008) Molecular graphics: *PLATON* (Spek, 2009) Software used to prepare material for publication: *publCIF* (Westrip, 2010)

### Crystal data

**Table:**

C_19_H_19_ClO_5_	*F*(000) = 1520
*M**_r_* = 362.79	*D*_x_ = 1.359 Mg m^−^^3^
Orthorhombic, *P**b**c**n*	Melting point: 383 K
Hall symbol: -P 2n 2ab	Mo *K*α radiation, λ = 0.71073 Å
*a* = 14.2134 (4) Å	µ = 0.24 mm^−^^1^
*b* = 10.2802 (2) Å	*T* = 293 K
*c* = 24.2786 (7) Å	Plate, yellow
*V* = 3547.51 (16) Å^3^	0.32 × 0.21 × 0.07 mm
*Z* = 8	

### Data collection

**Table:**

Bruker Kappa APEXII CCD diffractometer	*R*_int_ = 0.040
Graphite monochromator	θ_max_ = 25°, θ_min_ = 2.2°
scan	*h* = −16→16
34815 measured reflections	*k* = −12→12
3135 independent reflections	*l* = −28→28
2399 reflections with *I* > 2σ(*I*)	

### Refinement

**Table:**

Refinement on *F*^2^	Primary atom site location: structure-invariant direct methods
Least-squares matrix: full	Secondary atom site location: difference Fourier map
*R*[*F*^2^ > 2σ(*F*^2^)] = 0.034	Hydrogen site location: inferred from neighbouring sites
*wR*(*F*^2^) = 0.098	H atoms treated by a mixture of independent and constrained refinement
*S* = 1.05	*w* = 1/[σ^2^(*F*_o_^2^) + (0.0439*P*)^2^ + 1.2812*P*] where *P* = (*F*_o_^2^ + 2*F*_c_^2^)/3
3135 reflections	(Δ/σ)_max_ < 0.001
266 parameters	Δρ_max_ = 0.16 e Å^−^^3^
0 restraints	Δρ_min_ = −0.21 e Å^−^^3^

### Special details

**Table:**

Geometry. All e.s.d.'s (except the e.s.d. in the dihedral angle between two l.s. planes) are estimated using the full covariance matrix. The cell e.s.d.'s are taken into account individually in the estimation of e.s.d.'s in distances, angles and torsion angles; correlations between e.s.d.'s in cell parameters are only used when they are defined by crystal symmetry. An approximate (isotropic) treatment of cell e.s.d.'s is used for estimating e.s.d.'s involving l.s. planes.
Refinement. Refinement of *F*^2^ against ALL reflections. The weighted *R*-factor *wR* and goodness of fit *S* are based on *F*^2^, conventional *R*-factors *R* are based on *F*, with *F* set to zero for negative *F*^2^. The threshold expression of *F*^2^ > σ(*F*^2^) is used only for calculating *R*-factors(gt) *etc*. and is not relevant to the choice of reflections for refinement. *R*-factors based on *F*^2^ are statistically about twice as large as those based on *F*, and *R*- factors based on ALL data will be even larger.

### Fractional atomic coordinates and isotropic or equivalent isotropic displacement parameters (Å^2^)

**Table:**

	*x*	*y*	*z*	*U*_iso_*/*U*_eq_	
C18	−0.04648 (18)	−0.0556 (2)	0.06732 (9)	0.0670 (7)	
H183	0.004	−0.1085	0.0536	0.072 (7)*	
H182	−0.0823	−0.0222	0.037	0.087 (8)*	
H181	−0.0865	−0.1073	0.0905	0.077 (8)*	
Cl1	0.45882 (4)	0.06221 (6)	0.43367 (2)	0.06047 (19)	
O1	0.44408 (10)	0.21636 (16)	0.53159 (6)	0.0580 (4)	
O2	0.29752 (10)	0.35298 (14)	0.56689 (5)	0.0591 (4)	
O5	−0.00877 (10)	0.04997 (13)	0.09843 (5)	0.0520 (4)	
C2	0.36303 (12)	0.21629 (17)	0.50199 (7)	0.0392 (4)	
O4	0.04678 (10)	0.24539 (12)	0.15426 (6)	0.0536 (4)	
C9	0.20959 (14)	0.06296 (18)	0.33195 (7)	0.0418 (4)	
O3	0.26778 (11)	−0.12586 (15)	0.28885 (6)	0.0667 (4)	
C1	0.35824 (12)	0.14541 (16)	0.45372 (7)	0.0381 (4)	
C12	0.12393 (13)	0.10618 (18)	0.22209 (7)	0.0379 (4)	
C14	0.03967 (12)	0.01980 (18)	0.14509 (7)	0.0399 (4)	
C6	0.27698 (13)	0.14201 (16)	0.42094 (7)	0.0385 (4)	
C16	0.11233 (13)	−0.12733 (17)	0.21072 (7)	0.0422 (4)	
C10	0.21200 (13)	−0.03516 (17)	0.28728 (7)	0.0419 (4)	
C11	0.14658 (12)	−0.02000 (17)	0.23960 (7)	0.0384 (4)	
C3	0.28456 (13)	0.28768 (17)	0.51852 (7)	0.0415 (4)	
C8	0.27240 (14)	0.05921 (18)	0.37199 (7)	0.0428 (4)	
C15	0.05818 (13)	−0.10426 (19)	0.16356 (8)	0.0441 (5)	
C5	0.20091 (15)	0.21590 (19)	0.43854 (8)	0.0480 (5)	
C4	0.20394 (14)	0.28813 (19)	0.48690 (8)	0.0481 (5)	
C13	0.07162 (13)	0.12647 (17)	0.17529 (7)	0.0391 (4)	
C7	0.2259 (2)	0.4344 (3)	0.58622 (10)	0.0790 (8)	
H72	0.2461	0.4764	0.6195	0.092 (8)*	
H73	0.1706	0.3836	0.5936	0.130 (13)*	
H71	0.2118	0.4989	0.5589	0.114 (11)*	
C17	0.0853 (2)	0.3566 (2)	0.17956 (12)	0.0850 (9)	
H171	0.0632	0.4332	0.161	0.091 (8)*	
H173	0.1527	0.353	0.1775	0.176 (18)*	
H172	0.0662	0.3595	0.2175	0.107 (11)*	
C19	0.12707 (18)	−0.26652 (19)	0.22824 (10)	0.0631 (6)	
H191	0.1854	−0.2979	0.2134	0.092 (9)*	
H192	0.0762	−0.3191	0.2148	0.086 (8)*	
H193	0.129	−0.2712	0.2677	0.099 (9)*	
H12	0.1442 (11)	0.1768 (17)	0.2420 (7)	0.035 (5)*	
H9	0.1571 (14)	0.1257 (18)	0.3312 (8)	0.052 (6)*	
H15	0.0328 (14)	−0.176 (2)	0.1440 (8)	0.055 (6)*	
H8	0.3199 (16)	−0.005 (2)	0.3690 (9)	0.068 (7)*	
H4	0.1507 (14)	0.3358 (18)	0.4991 (8)	0.049 (5)*	
H5	0.1437 (15)	0.2187 (18)	0.4170 (8)	0.056 (6)*	
H11	0.4370 (19)	0.264 (3)	0.5596 (11)	0.086 (9)*	

### Atomic displacement parameters (Å^2^)

**Table:**

	*U*^11^	*U*^22^	*U*^33^	*U*^12^	*U*^13^	*U*^23^
C18	0.0762 (17)	0.0730 (15)	0.0518 (13)	0.0048 (14)	−0.0219 (13)	−0.0163 (12)
Cl1	0.0484 (3)	0.0816 (4)	0.0514 (3)	0.0225 (3)	−0.0062 (2)	−0.0222 (3)
O1	0.0492 (9)	0.0793 (10)	0.0454 (8)	0.0208 (7)	−0.0142 (7)	−0.0228 (8)
O2	0.0545 (9)	0.0761 (10)	0.0468 (8)	0.0214 (7)	−0.0101 (6)	−0.0256 (7)
O5	0.0543 (8)	0.0603 (8)	0.0413 (7)	−0.0015 (7)	−0.0140 (6)	0.0000 (6)
C2	0.0390 (10)	0.0447 (10)	0.0340 (9)	0.0033 (8)	−0.0052 (8)	0.0005 (8)
O4	0.0685 (10)	0.0434 (7)	0.0490 (8)	0.0009 (7)	−0.0120 (7)	0.0060 (6)
C9	0.0465 (11)	0.0448 (10)	0.0340 (9)	0.0026 (9)	−0.0011 (8)	0.0000 (8)
O3	0.0803 (11)	0.0626 (9)	0.0573 (9)	0.0270 (8)	−0.0224 (8)	−0.0160 (7)
C1	0.0399 (10)	0.0391 (9)	0.0354 (9)	0.0046 (8)	0.0018 (8)	0.0004 (7)
C12	0.0414 (10)	0.0398 (10)	0.0326 (9)	−0.0027 (8)	0.0023 (8)	−0.0043 (8)
C14	0.0353 (9)	0.0516 (11)	0.0329 (9)	−0.0018 (8)	−0.0012 (8)	−0.0006 (8)
C6	0.0439 (10)	0.0367 (9)	0.0350 (9)	−0.0008 (8)	−0.0026 (8)	0.0010 (7)
C16	0.0442 (11)	0.0422 (10)	0.0402 (10)	−0.0001 (8)	−0.0003 (8)	−0.0013 (8)
C10	0.0469 (11)	0.0431 (10)	0.0357 (9)	0.0027 (9)	−0.0019 (8)	0.0002 (8)
C11	0.0404 (10)	0.0440 (10)	0.0307 (9)	0.0011 (8)	0.0037 (8)	−0.0004 (7)
C3	0.0452 (11)	0.0447 (10)	0.0346 (9)	0.0041 (8)	−0.0010 (8)	−0.0043 (8)
C8	0.0487 (11)	0.0424 (10)	0.0374 (9)	0.0017 (9)	−0.0022 (9)	−0.0010 (8)
C15	0.0454 (11)	0.0440 (11)	0.0429 (10)	−0.0038 (9)	−0.0037 (8)	−0.0065 (9)
C5	0.0437 (12)	0.0534 (11)	0.0471 (11)	0.0047 (9)	−0.0107 (9)	−0.0066 (9)
C4	0.0433 (11)	0.0534 (11)	0.0474 (11)	0.0111 (9)	−0.0028 (9)	−0.0090 (9)
C13	0.0419 (10)	0.0405 (10)	0.0350 (9)	0.0007 (8)	0.0035 (8)	0.0044 (8)
C7	0.0757 (19)	0.0977 (19)	0.0635 (15)	0.0395 (17)	−0.0135 (13)	−0.0370 (15)
C17	0.133 (3)	0.0413 (13)	0.080 (2)	−0.0037 (14)	−0.0335 (18)	0.0099 (12)
C19	0.0734 (17)	0.0438 (11)	0.0720 (16)	−0.0010 (11)	−0.0164 (13)	−0.0010 (10)

### Geometric parameters (Å, º)

**Table:**

C18—O5	1.427 (2)	C14—C13	1.395 (2)
C18—H183	0.96	C6—C5	1.389 (3)
C18—H182	0.96	C6—C8	1.463 (2)
C18—H181	0.96	C16—C11	1.395 (2)
Cl1—C1	1.7356 (18)	C16—C15	1.400 (3)
O1—C2	1.358 (2)	C16—C19	1.507 (3)
O1—H11	0.85 (3)	C10—C11	1.493 (2)
O2—C3	1.365 (2)	C3—C4	1.379 (3)
O2—C7	1.399 (2)	C8—H8	0.95 (2)
O5—C14	1.362 (2)	C15—H15	0.95 (2)
C2—C1	1.382 (2)	C5—C4	1.390 (3)
C2—C3	1.394 (2)	C5—H5	0.97 (2)
O4—C13	1.371 (2)	C4—H4	0.95 (2)
O4—C17	1.409 (3)	C7—H72	0.96
C9—C8	1.320 (3)	C7—H73	0.96
C9—C10	1.482 (3)	C7—H71	0.96
C9—H9	0.99 (2)	C17—H171	0.96
O3—C10	1.225 (2)	C17—H173	0.96
C1—C6	1.403 (2)	C17—H172	0.96
C12—C13	1.374 (2)	C19—H191	0.96
C12—C11	1.403 (2)	C19—H192	0.96
C12—H12	0.919 (17)	C19—H193	0.96
C14—C15	1.377 (3)		
			
O5—C18—H183	109.5	O2—C3—C4	126.10 (17)
O5—C18—H182	109.5	O2—C3—C2	113.49 (15)
H183—C18—H182	109.5	C4—C3—C2	120.40 (16)
O5—C18—H181	109.5	C9—C8—C6	127.66 (18)
H183—C18—H181	109.5	C9—C8—H8	116.4 (13)
H182—C18—H181	109.5	C6—C8—H8	115.9 (13)
C2—O1—H11	109.0 (18)	C14—C15—C16	121.88 (17)
C3—O2—C7	118.98 (16)	C14—C15—H15	119.1 (12)
C14—O5—C18	117.19 (16)	C16—C15—H15	119.1 (12)
O1—C2—C1	119.42 (16)	C6—C5—C4	121.87 (18)
O1—C2—C3	121.74 (15)	C6—C5—H5	120.4 (12)
C1—C2—C3	118.84 (16)	C4—C5—H5	117.8 (12)
C13—O4—C17	117.48 (16)	C3—C4—C5	119.61 (18)
C8—C9—C10	120.23 (18)	C3—C4—H4	119.4 (12)
C8—C9—H9	122.9 (11)	C5—C4—H4	121.0 (12)
C10—C9—H9	116.7 (11)	O4—C13—C12	125.64 (16)
C2—C1—C6	122.34 (16)	O4—C13—C14	114.92 (15)
C2—C1—Cl1	117.20 (14)	C12—C13—C14	119.43 (16)
C6—C1—Cl1	120.44 (13)	O2—C7—H72	109.5
C13—C12—C11	121.03 (17)	O2—C7—H73	109.5
C13—C12—H12	119.0 (10)	H72—C7—H73	109.5
C11—C12—H12	119.9 (10)	O2—C7—H71	109.5
O5—C14—C15	125.34 (16)	H72—C7—H71	109.5
O5—C14—C13	115.01 (16)	H73—C7—H71	109.5
C15—C14—C13	119.65 (16)	O4—C17—H171	109.5
C5—C6—C1	116.93 (16)	O4—C17—H173	109.5
C5—C6—C8	122.23 (17)	H171—C17—H173	109.5
C1—C6—C8	120.79 (16)	O4—C17—H172	109.5
C11—C16—C15	117.97 (16)	H171—C17—H172	109.5
C11—C16—C19	124.08 (17)	H173—C17—H172	109.5
C15—C16—C19	117.91 (17)	C16—C19—H191	109.5
O3—C10—C9	120.70 (17)	C16—C19—H192	109.5
O3—C10—C11	120.46 (16)	H191—C19—H192	109.5
C9—C10—C11	118.83 (16)	C16—C19—H193	109.5
C16—C11—C12	119.93 (16)	H191—C19—H193	109.5
C16—C11—C10	121.64 (16)	H192—C19—H193	109.5
C12—C11—C10	118.34 (16)		

### Hydrogen-bond geometry (Å, º)

Cg1 and Cg2 are the centroids of the C1–C6 and C11–C16 rings, respectively.

**Table:**

*D*—H···*A*	*D*—H	H···*A*	*D*···*A*	*D*—H···*A*
O1—H11···O4^i^	0.84 (3)	2.31 (3)	3.007 (2)	140 (3)
O1—H11···O5^i^	0.84 (3)	2.37 (3)	3.042 (2)	138 (2)
C8—H8···Cl1	0.95 (2)	2.62 (2)	3.044 (2)	107.9 (15)
C7—H72···*Cg*2^i^	0.96	2.92	3.720 (3)	142
C18—H181···*Cg*1^ii^	0.96	3.00	3.512 (2)	115

Symmetry codes: (i) −*x*+1/2, −*y*+1/2, *z*+1/2; (ii) *x*−1/2, *y*−1/2, −*z*+1/2.
